# Cost-effectiveness of a mental health drop-in centre for young people with long-term physical conditions

**DOI:** 10.1186/s12913-022-07901-x

**Published:** 2022-04-19

**Authors:** Harrison Clarke, Walter Morris, Matteo Catanzano, Sophie Bennett, Anna E. Coughtrey, Isobel Heyman, Holan Liang, Roz Shafran, Neha Batura

**Affiliations:** 1 Institute for Global Health, University College London, London, UK; 2 UCL Great Ormond Street Institute of Child Health, University College London, London, UK; 3 Psychological and Mental Health Services, Great Ormond Street Hospital for Children, NHS Foundation Trust, London, UK

**Keywords:** Cost-effectiveness analysis, Economic evaluation, Child and adolescent mental health, Long-term physical health

## Abstract

**Background:**

Paediatric patients being treated for long-term physical health conditions (LTCs) have elevated mental health needs. However, mental health services in the community are difficult to access in the usual course of care for these patients. The Lucy Project – a self-referral drop-in access point—was a program to address this gap by enrolling patients for low-intensity psychological interventions during their treatment for LTCs. In this paper, we evaluate the cost-effectiveness of the Lucy Project.

**Methods:**

Using a pre-post design, we evaluate the cost-effectiveness of the intervention by calculating the base-case incremental cost-effectiveness ratio (ICER) using outcomes data and expenses recorded by project staff. The target population was paediatric patients enrolled in the program with an average age of 9 years, treated over a time horizon of 6 months. Outcome data were collected via the Paediatric Quality of Life Inventory, which was converted to health utility scores using an instrument found in the literature. The QALYs were estimated using these health utility scores and the length of the intervention. We calculate a second, practical-case incremental cost-effectiveness ratio using streamlined costing figures with maximum capacity patient enrolment within a one-year time horizon, and capturing lessons learned post-trial.

**Results:**

The base-case model showed an ICER of £21,220/Quality Adjusted Life Years (QALY) gained, while the practical model showed an ICER of £4,359/QALY gained. The practical model suggests the intervention garners significant gains in quality of life at an average cost of £309 per patient. Sensitivity analyses reveal use of staff time was the greatest determinant of the ICER, and the intervention is cost-effective 75% of the time in the base-case model, and 94% of the time in the practical-case model at a cost-effectiveness threshold of £20,000/QALY gained.

**Conclusions:**

We find the base-case intervention improves patient outcomes and can be considered cost-effective according to the National Institute for Health and Care Excellence (NICE) threshold of £20,000—£30,000/QALY gained, and the practical-case intervention is roughly four times as cost-effective as the base-case. We recommend future studies incorporate a control group to corroborate the effect size of the intervention.

**Supplementary Information:**

The online version contains supplementary material available at 10.1186/s12913-022-07901-x.

## Background

Children and adolescents with long-term physical health conditions (LTCs) exhibit significantly elevated mental health needs [1–6]. The likelihood of developing a mental health disorder for children with LTCs is approximately three times greater than for children in the general population [7]. Co-existing mental health problems can also exacerbate pre-existing LTCs leading to poorer clinical outcomes [8–11]. Children with both physical and mental health conditions, therefore, face significantly lower quality of life compared to those with physical health problems alone.

The prevalence of comorbid physical and mental health conditions is rising in many countries, and its economic burden falls on patients, their families as well as health providers [12]. A longitudinal cohort analysis showed that the likelihood of hospitalizations for children with psychiatric diagnosis is five times greater compared to those without any mental health diagnosis. Further, the greatest growth in hospitalizations were observed for children with comorbid physical conditions in addition to co-existing psychiatric diagnosis amounting to 78% of all hospitalizations for children with existing mental health conditions [13]. The additional annual insurance payments associated with co-morbid mental health conditions in the US were estimated at US$8.8 billion; parents of children with LTCs and associated mental health conditions were burdened with 59% higher payments compared to parents of children with LTCs alone [14]. Increased rates of hospitalization pose a significant strain on the resources of healthcare providers and the patients. The burden on the healthcare provider may also include increased visits to the doctors and additional medication use for both physical and psychological conditions [15]. For the patient and the guardian, the economic burden may be realized in the form of out-of-pocket expenses for medication and other treatments, travel expenses and loss of income to the guardian depending on the extent of care required [16, 17]. Due to the patient’s long-term physical condition, these costs are likely prolonged and accumulate over the long term.

Early integration of mental and physical health services are priority areas for the National Health Service (NHS) [18]. Meta-analyses indicate that psychological interventions may reduce care costs by 20%; the resulting saving in physical care costs are likely to exceed any costs incurred for the delivery of the psychological treatment [19–21]. Yet, the current standard care for paediatric mental health in the UK is significantly limited due to the lack of routine integration with physical healthcare. The provision of paediatric psychology in the UK is typically to provide input when the problem is related to the LTC, for example difficulties adjusting to a diagnosis, procedural fears/phobias, or problems disclosing the diagnosis to peers [22]. Where there is a mental health problem that is not related to the LTC, a referral to local children and young people’s mental health services is made. Evidence of increasing demand for mental health support fuelled by the COVID-19 pandemic [23–27], coupled with the raised inclusion criteria, referrals for children with physical conditions to the child and adolescent mental health services (CAMHS) are at greater risk of being turned down with a tendency to focus solely on the existing physical health condition treated by other services [28]. Figures on national waiting times show that some young people are waiting up to 182 days from referral to the start of treatment [20]. As a result, even if young people are accepted after being referred to local CAMHS, they are often left untreated for long periods of time, unless problems become severe. This neglects the aforementioned negative clinical effects of mental health conditions in the context of an LTC.

The ‘Lucy Project’ aimed to fill this gap by testing and evaluating an easily accessible mental health intervention for children with LTCs and their families at Great Ormond Street Hospital [29, 30]. The project was distinguished by a proactive approach to patient enrolment, including dissemination of recruitment leaflets, referrals of patients to the project by physicians, signposting, and a booth physically located at the reception of the national paediatric hospital where patients and family members could drop in and enquire about enrolment and receive treatment. Upon consent, patients underwent a mental health evaluation process administered by the Lucy Project staff, which determined the most appropriate interventions for the patient to be allocated to. The intervention comprised of National Institute for Health and Care Excellence (NICE) recommended low-intensity psychological interventions, direction to self-help materials, neurodevelopmental assessment and referral to appropriate internal/external services. The treatments allocated were based on a diagnostic formulation using an abbreviated, self-help version of a modular psychological intervention MATCH-ADTC [31]. For example, the abbreviated, self-help version of the module of MATCH addressing anxiety was offered when generalized anxiety was the primary presenting problem.

The mental health outcomes of the Lucy Project were measured based on parent-reported Strengths and Difficulties Questionnaire (SDQ) and Paediatric Quality of Life Inventory (PedsQL), which were administrated at baseline and six months post-baseline. The trial outcome was associated with a significant improvement in the outcome scores suggesting that this transdiagnostic treatment can reduce emotional and behavioural symptoms and improve quality of life in children with long-term physical condition. More detailed findings of the evaluation are presented in the paper by Catanzano [29]. The objective of this paper is to estimate the costs and cost effectiveness of the intervention to informing important resource allocation decisions for these crucial services.

## Methods

In this retrospective study, intervention costs and outcomes were estimated from the trial to assess the incremental cost effectiveness ratio (ICER) of a brief transdiagnostic psychological treatment for the paediatric population with long-term physical conditions compared to standard care. It is important to clarify that due to the complexity in quantifying standard care costs, coupled with the previously noted tendency to focus solely on the physical conditions of these patients, the alternative scenario is one where the incremental costs and effects are assumed to equal zero. In other words, the intervention is assessed based on the cost effectiveness analysis starting from the origin of the cost effectiveness plane [32]. Two models were developed to conduct the study. The base-case model estimated the ICER using observed costs and outcomes from the trial. The second model was a streamlined practical model incorporating efficiencies drawing on insights gained from the Lucy trial as advised by the clinical team.

The study is conducted in the context of a research study in the NHS in the UK and reports the cost-effectiveness from the perspective of the health provider. Currently, children with long-term physical illnesses are admitted to standard care through a process of referral/self-referral to CAMHS. As such, this study assesses the resulting ICER of the Lucy trial against the NICE specified threshold of £20,000-£30,000 per Quality Adjusted Life Years (QALY) gained [33]. Collected costs and outcomes ranged from 2019–2020 for a time horizon of 12 months based on the duration of the study. To be conservative, costs were estimated using inner London rates and valued in 2020 pound sterling (£).^1^ Cost and outcomes were discounted at a rate of 3.5% [35].

### Trial design, outcome measures and utility weights

The Lucy Project was conducted in two phases. Recruitment for the pilot phase started in January 2018 and ended in December 2018 [30]. The main trial phase started recruitment in January 2019 and ended in December 2019. The pilot phase was conducted to estimate recruitment and attrition; the purpose of the subsequent main trial was to gather preliminary evidence of effectiveness.

All outcome measures were completed at baseline upon consent and at 6 months from baseline. The pilot phase was measured only using the SDQ [36] while the PedsQL [37] was added for the main trial. The 23 items in the PedsQL comprise four subscales: physical, emotional, social and school functioning. Two summary scores can be computed: the psychosocial and total score. The PedsQL psychosocial score is calculated from three subscales which measure emotional, social, and school functioning. The PedsQL total score is the mean of all items. PedsQL places greater emphasis on quality of life and functionality, as opposed to the SDQ, which primarily measures symptom severity. This is supported by previous research indicating that patients place importance on functioning in aspects of life that map well onto the PedsQL subscales – particularly among young people with LTCs [38–40]. As such, this study reports the ICER derived from the costs and outcomes of the main trial phase using PedsQL scores as the measure of outcome. For the purpose of the cost-effectiveness analysis, the pre- and post-intervention PedsQL total scores were converted to health utilities measured by the EuroQol-5D quality of life instrument using a model developed by Khan, et. al. [41]. The trial did not collect data beyond the 6-month time horizon for each patient. Therefore, we assume that the QALYs at 6-months will be felt for another 6 months (one year in total) [42].

A detailed summary of patient demographics for both trials can be found in Additional file 1: Appendix A. The study participants had to be a patient at the paediatric hospital for a physical health condition within the last 6 months or be a caregiver/family member/sibling of such a patient. Further, patients had to exhibit common mental health needs such as anxiety, depression and/or behavioural difficulties which were not currently being treated by the paediatric psychology services.^2^ Based on the initial triage assessment, patients were allocated to an intervention based on a clinical decision-making algorithm considering key mental health factors [29]. Table 1 summarizes participant allocation in the main trial, which included low intensity cognitive behavioural therapy (CBT), direction to self-help materials, neurodevelopmental assessment and referral to appropriate internal/external services. The low intensity CBT was delivered by the Lucy Project team; patients receiving other treatments were referred on to specialist services and were treated accordingly.

**Table 1:** Participant allocations by intervention

Interventions	Proportion of Participant Allocations: Main Trial
Low Intensity CBT	31%
Referral	42%
Neurodevelopmental assessment	1%
Signposting to resources only	26%

### Resource use and costs

Counts of resource use were directly drawn from line-item expense figures recorded in the Lucy Project budget, as reported by the clinical team. Expense figures were denominated in pounds sterling. Costs were categorized into start-up and implementation costs which were further sectioned into staff, capital, overhead and other costs; this was measured in terms of expected unit cost per patient. Cost incurred by external services where patients were referred on was not included in this study.^3^

For the base-case model, all costs reported in the Lucy trial were collected and separated into research and implementation costs based on input by the clinical team. Overhead costs were estimated using publicly available information from the GOSH Annual Report [43], the Office for National Statistics [44] and the Greater London Authority Economics Report [45]. Ratios between property prices and rent in central London were used to estimate the rent of the booth space and office spaces for staff. Some start-up costs and capital costs (such as the cost of the booth purchase, laptops and mobile phones) were carried over from the pilot phase and included in the cost of the main trial. Total estimated costs were divided by the total number of patients analysed for the outcome (*n* = 93).

Following NICE commissioning guidance and discussion with key intervention staff, the practical model was set up based on the assumption that a trained Psychological Wellbeing Practitioner (PWP) can see up to 200 patients in a single year^4^ [46]. Estimated costs were derived from the 2019–2020 AfC pay scales for the NHS [47] using inner London rates. The clinical psychologist’s time was doubled from the base-case model to account for their leading role in allocating patients to the appropriate treatment. Conversely, the psychiatrist’s time was reduced to reflect their limited involvement in supporting the clinical psychologist with patient allocations. Finally, the drop-in booth and related costs were removed. The booth was installed to serve a dual purpose of recruiting and offering a place for patients to receive treatment. Very few patients chose to receive treatment in the booth as it was placed in a busy reception area in order to gain exposure as a recruitment tool. For this reason, the booth costs were replaced with cheaper alternative recruitment materials including posters and leaflets. Finally, as the practical model assumes that all patients are seen within the single year of 2019, no discount was applied on the costs and outcomes. In both the base-case and practical model, screening costs are captured in the costs for the initial session for each patient, where the course of treatment is decided.

### Simulations and sensitivity analyses

Several sensitivity analyses were conducted to evaluate the impact of varying baseline estimates for the base-case and theoretical models. These included varying: 1) staff costs; 2) overhead costs; 3) capital costs; 4) patient counts; 5) expected outcomes; 6) length of the study.^5^ Parameters (1-3) was varied by ± 20% to assess how different cost categories influence the outcome. Patient count was increased to 200 for the base-case model and reduced to 93 for the practical model. These changes effectively reverse the patient count between the two models in order to assess the resulting effect on the ICERs. As the clinical outcome is based on a single trial, the expected outcome reported in QALYs were varied by ± 20% to assess the impact on the ICERs. Finally, the length of the study was extended to 1.5 years for the practical model so that it aligns with the base-case model. This was done to assess how the two models compare when it has the same time span.

We also conducted a probabilistic sensitivity analysis (PSA) using a Monte-Carlo Simulation technique sampling the costs and outcomes simultaneously from their observed distributions. Patient level outcome data from the main Lucy trial was used to assess the underlying distribution of the outcome. To assess the cost distribution, per patient costs were estimated based on the treatment allocations and the number of treatment sessions.^6^ To increase sample size and the accuracy, per patient cost from the main and pilot phase was used to determine the distribution of patient-level costs and outcomes. Based on the modelling analyses, a normal distribution was fitted for the outcome and a beta distribution was used for the costs. A total of 20,000 simulations were conducted (equivalent of 200 patients for 100 different sites) for both models. For the theoretical model, the distribution from the main analysis was applied by adjusting for the difference in the expected per-patient costs. Finally, the results from the PSA were used to derive cost-effectiveness acceptability curves (CEAC) for both models.

All analyses were conducted using R-4.0.2^7^ and Excel 2019.

Ethics approval was granted by the London Riverside Research Ethics Committee (REC reference number: 16/LO/1915).

## Results

The undiscounted trial outcome summarized in Table 2 reports an improvement in the participants’ quality of life measured in PedsQL total score (mean increase of 7.5). All subscales, except for physical health, indicated a statistically significant improvement. The undiscounted expected incremental gain was estimated at 0.071QALYs using the algorithm developed by Khan, et. al. [41].

**Table 2:** Comparison of PedsQL scores at baseline and at 6 months follow-up

		Pre	Post		
**PedsQL Measures**	**n**	**Mean (SE)**	**Mean (SE)**	**Mean difference (CI)**	***P* value**
Physical Health	93	58.60 (3.24)	62.18 (3.06)	− 3.58 (− 7.51 to 0.34)	0.075
Psychosocial Health	93	51.98 (2.10)	59.81 (2.03)	− 7.83 (− 11.25 to − 4.42)	< 0.001***
Emotional Functioning	93	49.61 (2.46)	56.16 (2.53)	− 6.54 (− 12.21 to − 0.87)	0.024*
Social Functioning	93	54.88 (3.04)	64.84 (2.73)	− 9.96 (− 15.58 to − 4.33)	0.001**
School Functioning	93	51.45 (2.51)	58.44 (2.37)	− 6.99 (− 11.41 to − 2.58)	0.002**
**PedsQL total score**	**93**	**54.38 (2.31)**	**61.88 (2.39)**	** − 7.50 (− 10.45 to − 4.55)**	< 0.001***
**PedsQL total to QALY**	**93**	**0.6577**	**0.7286**	**0.0709**	

*p < 0.05, **p < 0.01, ***p < 0.001

*n* = total cases analyzed for the main trial

*CI* Confidence Intervals

*PedsQL* Pediatric Quality of Life Inventory

*QALY* Quality Adjusted Life Years

Multiple imputation techniques with fully conditional specifications were used to account for missing data

### Base-case

Undiscounted total intervention costs of the Lucy project amounted to £138,100. Estimated start up and implementation costs were £23,000 (17%) and £115,000 (83%) respectively. Based on 93 patients who were analysed in the outcome for the Lucy trial, average intervention costs per patient were estimated at £1,500. As summarized in Table 3, staff costs accounted for 74.2% of the costs while capital, overhead and other costs (i.e., recruitment materials) accounted for 16.3%, 9.1% and 0.4% respectively.

**Table 3:** Estimated cost parameters: base-case model

Parameter	Per-Patient Estimate (£)	Source
**Base-Case Model (*n* = 93)**		
**Staff Costs—Total**	**1112.93**	
Clinical Psychologist	27.92	Project Finance Report
Psychological Wellbeing Practitioner (PWP)	395.74	Project Finance Report
Admin Support	325.37	Project Finance Report
Booth Volunteers	120.47	Project Finance Report
Child and Adolescent Psychiatrist	240.46	Project Finance Report
Other Staff Costs	2.98	Project Finance Report
**Capital Costs—Total**	**244.95**	
Booth Hire & Purchase	218.69	Project Finance Report
Equipment	14.71	Project Finance Report
Other Capital Costs	11.54	Project Finance Report
**Overhead Costs—Total**	**136.18**	
Staff Overhead Costs	103.16	GOSH Annual Report ONS GLA
Booth Overhead Costs	33.02	GOSH Annual Report ONS GLA
**Other Costs—Total**	**5.52**	Project Finance Report
**Total Cost Per Patient**	**1499.59**	

Signposting to resources was generally carried out by including these in the GP letter that patients received following their assessment, so no extra costs were incurred. For costs of neurodevelopmental assessments carried out by the team, these were already included within existing staff costs

After applying a 3.5% discount rate, the base-case incremental gain was 0.0698 QALYs and incremental costs were £1,482 per patient. Combining these estimates resulted in ICER of £21,200 per QALY (Table 4).

**Table 4:** Summary of cost-effectiveness: base-case model

Scenarios	Incremental Costs (£)	Incremental Effects (QALY)	ICER (CI)
Undiscounted	£1,499.59	0.0709	£21,147
Discounted	£1,482.09	0.0698	£21,220 (-8,848, 74,831)

**Fig. 1:**
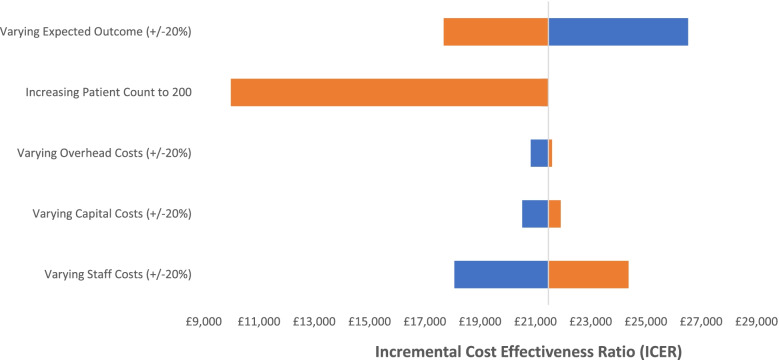
Base-Case Model Tornedo Plot of Multiple One-Way Sensitivity Analyses

The one-way sensitivity analyses summarized in the form of a tornado plot indicates that varying the overhead and capital cost has little impact on the base-case ICER (Fig. 1). Conversely, changes to the staff costs lead to a more substantial fluctuation in the resulting ICER. Varying the expected outcome by ± 20% also lead to a significant variation in the ICER with greater sensitivity associated to reductions in the expected QALY gained. A 20% increase to the expected QALY gained reduced the ICER by 16.5% (£3,500) while reduction of 20% in the expected QALY gained increased the ICER by 25% (£5,300). Finally, increasing the patient count to 200 had the greatest effect whereby the resulting ICER reduced by 53% (£11,200).

**Fig. 2:**
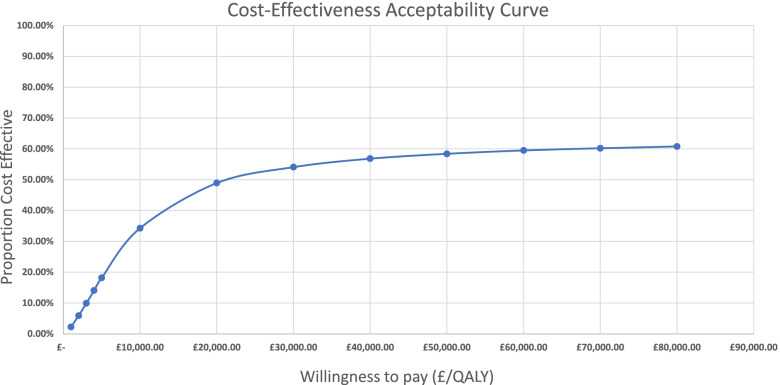
Base-case Model Cost-Effectiveness Acceptability Curve

Figure 2 represents the CEAC derived from the Monte-Carlo simulations for the base-case model. The results indicate that over 49% of all simulated outcomes were cost-effective with a willingness to pay of £20,000/QALY. This increased to 54% with a willingness to pay of £30,000/QALY and gradually converges towards 65% as the willingness to pay increases. Based on this simulation, 35% of the simulated outcomes featured QALYs lost by patients on average, which would not be considered cost-effective under any decision rule (see north west quadrant of the ICER scatter plots presented in Additional file 1: Appendix B). Accounting for outcomes with QALYs gained only, 75% and 83% of the simulated outcomes would be considered cost-effective at a willingness to pay of £20,000/QALY and £30,000/QALY respectively.

### Practical model

Total intervention costs for the practical model were estimated to be £61,800; start up and implementation costs were £4,000 (6.5%) and £57,800 (93.5%) respectively. The expected intervention costs per patient were derived assuming 200 enrolled patients which is in accordance with the annual number of patients that can be seen by a single PWP. Resulting average intervention costs per patient were estimated at £309 (Table 5). Staff costs accounted for 79.3% of the costs while capital, overhead and other costs accounted for 4.6%, 14.5% and 1.6% respectively.

**Table 5:** Estimated cost parameters: practical model

Parameter	Per-Patient Estimate (£)	Source
**Practical Model (*n* = 200)**		
**Staff Costs—Total**	**244.99**	
Clinical Psychologist	14.62	AfC Pay Scales
Psychological Wellbeing Practitioner (PWP)	158.93	AfC Pay Scales
Admin Support	56.15	AfC Pay Scales
Booth Volunteers	0.00	
Child and Adolescent Psychiatrist	15.30	AfC Pay Scales
Other Staff Costs	0.00	
**Capital Costs—Total**	**14.25**	
Booth Hire & Purchase	0.00	
Equipment	6.84	Project Finance Report
Other Capital Costs	7.41	Project Finance Report
**Overhead Costs—Total**	**44.81**	
Staff Overhead Costs	44.81	GOSH Annual Report ONS GLA
Booth Overhead Costs	0.00	
**Other Costs—Total**	**5.05**	Project Finance Report
**Total Cost Per Patient**	**309.09**	

No discount was applied to the practical model as all outcomes were assumed to be from a single year in 2019. The ICER from the practical model resulted in £4,400 per QALY as summarized in Table 6.

**Table 6:** Summary of cost-effectiveness: practical model

Scenarios	Incremental Costs (£)	Incremental Effects (QALY)	ICER (CI)
Undiscounted	£309.09	0.0709	£4,359 (-8,862, 6,878)

**Fig. 3:**
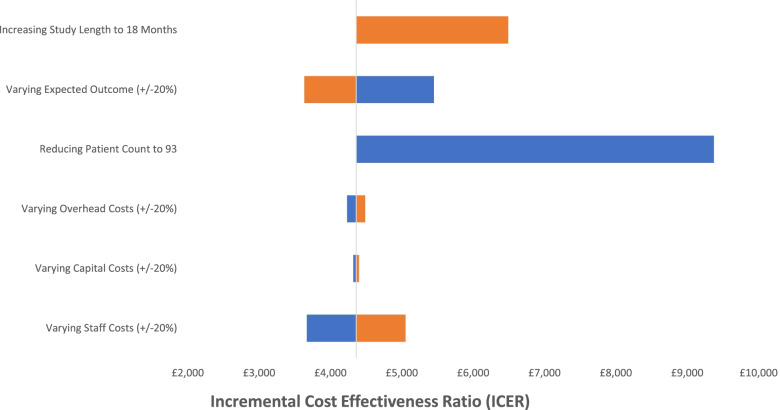
Practical Model Tornedo Plot of Multiple One-Way Sensitivity Analyses

Similar to the base-case model, the one-way sensitivity analyses summarized in Fig. 3 shows significant variations associated with staff costs, however little impact was recorded from changes to overhead and capital costs. Reducing the patient count to 93 had the most significant impact which resulted in an ICER increase of 115% (£5,000). A 20% increase to the expected QALY gained reduced the ICER by 17% (£730) while reduction of 20% in the expected QALY gained increased the ICER by 25% (£1,100). Finally, increasing the study length of the practical model to 18 months (equivalent study duration to the base-case model) increased the ICER by 49% (£2,100).^8^

**Fig. 4:**
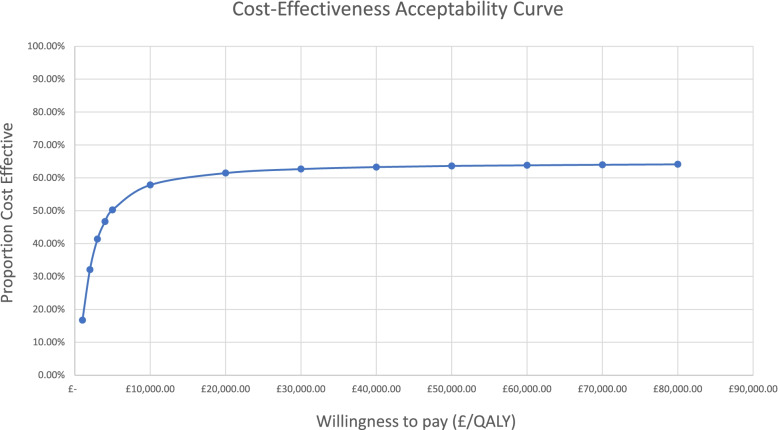
Practical Model Cost-Effectiveness Acceptability Curve

The CEAC in Fig. 4 shows that 61% of the outcomes were cost-effective at willingness to pay of £20,000/QALY which increased to 63% at willingness to pay of £30,000/QALY. Similar to the base-case model, 35% of all simulated outcomes featured negative QALY (Additional file 1: Appendix C). Therefore, accounting the outcomes with positive QALY only, this proportion increased to 94% and 96% at a willingness to pay of £20,000/QALY and £30,000/QALY respectively.

## Discussion

This study assessed the costs and cost effectiveness of a brief transdiagnostic psychological assessment and treatment for children’s mental health needs in the context of LTCs. The intervention provided low-intensity CBT as well as referrals to other services based on individual assessments. The Lucy Project offers an expected ICER of £21,200, which can be considered cost-effective according to the current NICE cost-effectiveness threshold of £20,000 to £30,000 per QALY conditional to meeting certain factors specified by NICE [48] [49].

The expected cost per patient in the base-case model depends significantly on the total number of patients in the study. When the patient count increases to 200 in the base-case model (Fig. 1), the expected ICER falls to £10,000, indicating a significant scale effect that could be realized from optimal use of staff time and the diminished per-patient contributions to fixed costs. The practical model was therefore built on the assumption of having a single PWP treating 200 patients within the single year 2019, in accordance with the NICE guidelines. This adjustment alone addresses the inefficiencies associated with staff utilization by employing the PWP to capacity. Additionally, the practical model also included adjustments which further reduced the costs and improved the resulting ICER. These adjustments were made based on future best practices from the clinical team as outlined in the methods section. The practical model does not change who is delivering the intervention and therefore there is no reason utility gains or recruitment should be compromised. Data from the pilot year suggests that only 12.5% of the participants were recruited via ‘drop-in’ to the booth [30]. A similar number were signposted by clinicians, which would still occur even without the booth. The majority (73.4%) were recruited via booth volunteers handing out leaflets, which could still occur in the practical model. Although, booth volunteers were not costed in the practical model (as they were hospital volunteers, no extra cost was incurred), varying staff costs by + 20% would increase the ICER to approximately £5000 (which is still below the NICE cost-effectiveness threshold of £20,000-£30,000/QALY) as shown in the sensitivity analysis in Fig. 4. The same sensitivity analysis shows that even if there were to be a reduction of 20% in the expected QALY gained with the practical model, the ICER would only increase to £5,459, which remains well below the NICE cost-effectiveness threshold of £20,000/QALY. The average total cost per patient for this model was estimated at £309 which is comparable to the cost per patient of £343 (ICER £5,374) reported in a larger study assessing the cost-effectiveness of CBT [50]. This model therefore offers a sensible estimate to assess the cost-effectiveness of the intervention from the perspective of the healthcare system and indicates a significantly improved ICER of £4,400 per QALY. Should the intervention be considered for a wider implementation, efficient use of resources (particularly regarding the PWP seeing the optimal number of patients) would have significant implication on the resulting ICER and there are reasons to believe that this is plausible. Due to the effect of the COVID-19 pandemic, many of the treatment sessions for the Lucy Project were successfully carried out remotely. This allows flexibility to hire and allocate key resources regardless of their location, which could greatly support the efficient allocation of resources.

From the one-way sensitivity analyses (Figs. 1 and 4), variation in staff cost showed a significant impact on the resulting ICER for both models, while changes to capital and overhead costs had little implication to the cost-effectiveness of the study. Given that the staff costs represented the majority of the costs (74.2% and 79.3% in the base-case and practical model respectively), the outcome of the sensitivity analyses is to be expected. As the study outcome was estimated from a single study, resulting ICERs were assessed against varying levels of outcome. Although the ICERs were sensitive to these changes, neither models exceeded £30,000 per QALY indicating that the intervention is likely to fall within the cost-effective threshold. For the practical model, the study duration was increased to 18 months to be in line with the duration of the intervention. This increased the ICER in the practical model to £6,500 which is still considerably below the defined ICER threshold.

The probabilistic sensitivity analyses show that the proportion of cost-effective outcomes converge to 65%. This is due to the proportion of simulations with negative outcomes that fell in the northwest quadrant of the cost-effectiveness plane (Additional file 1: Appendix B and C). The substantial proportion of outcomes that fall in this quadrant are likely explained by the fact that outcomes were fitted to a normal distribution. Given that the mean QALYs gained in the trial was small, a fraction of simulation results showed negative outcomes. While it seems unlikely that this intervention would yield a negative change in QALYs, it cannot be said for certain that it wouldn’t due to our lack of a control group, which limits the certainty of our results. Nonetheless, since it is also a distinct possibility that the QALYs lost in the simulation could be attributed to the outcomes being drawn from a normal distribution with a mean close to zero, we saw it fit to calculate the proportion without the negative QALY outcomes. Based on this, the proportion of cost-effective outcomes increases to 75% for the base-case and 94% for the practical model at a £20,000 willingness to pay. This further increased to 83% and 96% with a willingness to pay of £30,000.

The overall estimated ICERs from the two models differ significantly (£16,800). The base-case model captures the upper spectrum of the cost-effectiveness range, while the practical model offers an indication of the interventions’ cost-effectiveness conducted in an efficiently streamlined setting. Despite uncertainties, there are reasons to believe that an efficient model can be achieved within the NHS. For instance, majority of the intervention was delivered remotely indicating that effective CBT can be administered without in-person sessions. In a scaled-up program implementing best practices, a single PWP could treat patients recruited from multiple sites remotely, which would allow PWPs to maximize patients treated to capacity, while spreading costs between several providers, reducing costs for each. Furthermore, costs on recruitment tools could be reduced if it is made standard practice for physicians to refer patients to a program like the Lucy Project based on their eligibility criteria, possibly as part of a hospital-wide screening program [51]. Overall, despite the base-model ICER resulting above the NICE cost-effectiveness threshold of £20,000 per QALY, preliminary evidence from the theoretical model indicates that the true ICER is likely to be below this threshold and, potentially, as low as £4,400 per QALY.

The absence of a control group is a limitation of the study as noted in the clinical paper by Catanzano [29]. For example, a lack of control group makes it difficult to know whether the effects are specific to the intervention or just a product of other confounding variables, such as time. Before and after studies are at very high risk of bias due to 'regression to the mean', and this is particularly true for mental health conditions that can vary substantially over relatively short periods of time. We attempted to mitigate this risk, by not restricting inclusion into the study to individuals above threshold on the total score or any particular subscale of the SDQ or PEDSQL. Nevertheless, the small to moderate effect sizes found in our study were similar to the meta-analysis by Bennett and colleagues (2019), who found that the effect size of self-help and guided self-help on symptoms of common mental health disorders when compared to a control group (including: waiting list, attention and nonactive treatment as usual) was g = 0.49 (*n* = 44; 95% CI: 0.37 to 0.61, *p* < 0.01 [52]. Furthermore, the pragmatic design of this analysis lends weight to the effectiveness of the intervention in an uncontrolled environment [53]. The Lucy Project was piloted within a hospital setting and was designed to be easily integrated into existing care practices, therefore these results can be generalized to care facilities similar to Great Ormond Street Hospital [54]. Therefore, if the effect of the intervention on patient outcomes can be corroborated by a trial with a control group, it would bode well for the adoptability of the intervention into standard care procedures for chronically ill paediatric patients.

It is possible that a degree of cost-shifting occurred [55], especially for those patients who were referred (e.g., costs to local CAMHS were not included as service use data was not collected). However, it is equally possible that in a subset of those receiving low-intensity CBT, this input was sufficient and therefore involvement of other services e.g., CAMHS, was avoided, thereby reducing costs to the healthcare system. This would need to be tested in a randomized trial where service use data was collected.

We acknowledge that the absence of costs incurred by external services is a limitation of the study. Because patients who were referred to other providers were not referred to a single external service, the operating costs of the external providers are likely highly varied and not subject to estimation given the limited scope of the study. However, as the present analysis is conducted from the provider perspective, and an individual service provider would not incur costs for treatment after a referral, the analysis is still useful at the facility level.

## Conclusions

The study suggests that, on its own, compared to a scenario where the incremental costs and effects would be equal to zero, this intervention is associated with positive health outcomes at a cost that can be considered cost-effectiveness subject to conditions. Based on the practical model, the brief interventions delivered in this project are associated with significant gains in quality of life at an average cost of £309 per patient. The primary limitation of this study is the lack of control group which may affect the reliability of the estimated costs and effects. In particular, the effects of the intervention cannot be entirely separated from the effects of time and/or treatment undergone for the physical conditions. The results shown in this paper are suggestive of the cost-effectiveness of the intervention, however, it is recommended that future studies be conducted using an RCT design to better address possible confounders and establish more reliable estimates of both costs and outcomes.

## Supplementary Information

**Additional file 1: Appendix A.** Patient Characteristics from the OutcomeStudy. **Appendix B**. Base-case Model ICER Scatter Plot. **Appendix C**. Practical Model ICER Scatter Plot.

## Data Availability

The datasets used and/or analysed during the current study are available from the corresponding author on reasonable request.

## Abbreviations

CAMHS: Child and adolescent mental health services
CBT: Cognitive behavioural therapy
CEAC: Cost-effectiveness acceptability curves
GOSH: Great Ormond Street Hospital
ICER: Incremental cost-effectiveness ratio
LTC: Long-term physical health conditions
NHS: National Health Service
NICE: National Institute for Health and Care Excellence
PedsQL: Paediatric Quality of Life Inventory
PSA: Probabilistic sensitivity analysis
PWP: Psychological Wellbeing Practitioner
QALY: Quality Adjusted Life Years
SDQ: Strengths and Difficulties Questionnaire
UK: United Kingdom
CAMHS: Child and adolescent mental health services
CBT: Cognitive behavioural therapy
CEAC: Cost-effectiveness acceptability curves
GOSH: Great Ormond Street Hospital

## Footnotes

1 Rates were inflated using Consumer Price Index (CPI) available from the Office for National Statistics [34].(ONS) OfNS. Consumer price inflation time series (MM23). 2021.

2 See the clinical paper by Catanzano et al. [29] for more detail on the inclusion criteria.

3 This includes costs that were incurred by the healthcare system after being allocated to one of the following interventions; direction to self-help materials, neurodevelopmental assessment and referral to appropriate internal/external services.

4 The actual number estimated was 213 patients—we assumed 200 to be conservative.

5 Length of the study was varied only for the theoretical model.

6 Initial assessment used to determine patient allocation was included in the treatment sessions.

7 Mainly used for modelling the distributions.

8 Costs were inflated to 2020 valuation and a discount of 3.5% were applied.

## References

[1] Adams JS, Chien AT, Wisk LE (2019). Mental illness among youth with chronic physical conditions. Pediatrics.

[2] Moore DA, Nunns M, Shaw L, Rogers M, Walker E, Ford T (2019). Interventions to improve the mental health of children and young people with long-term physical conditions: linked evidence syntheses. Health Technol Assess.

[3] Pinquart M (2020). Posttraumatic stress symptoms and disorders in children and adolescents with chronic physical illnesses: a meta-analysis. J Child Adolesc Trauma.

[4] Pinquart M, Shen Y (2011). Anxiety in children and adolescents with chronic physical illnesses: a meta-analysis. Acta Paediatr.

[5] Pinquart M, Shen Y (2011). Behavior problems in children and adolescents with chronic physical illness: a meta-analysis. J Pediatr Psychol.

[6] Pinquart M, Shen Y (2011). Depressive symptoms in children and adolescents with chronic physical illness: an updated meta-analysis. J Pediatr Psychol.

[7] Blackman JA, Gurka MJ, Gurka KK, Oliver MN (2011). Emotional, developmental and behavioural co-morbidities of children with chronic health conditions. J Paediatr Child Health.

[8] Chavira DA, Garland AF, Daley S, Hough R (2008). The impact of medical comorbidity on mental health and functional health outcomes among children with anxiety disorders. J Dev Behav Pediatr.

[9] Cottrell D (2015). Prevention and treatment of psychiatric disorders in children with chronic physical illness. Arch Dis Child..

[10] Kovacs M, Mukerji P, Drash A, Iyengar S (1995). Biomedical and psychiatric risk factors for retinopathy among children with IDDM. Diabetes Care.

[11] Lustman PJ, Clouse RE (2005). Depression in diabetic patients: the relationship between mood and glycemic control. J Diabetes Complications.

[12] Sartorious N (2013). Comorbidity of mental and physical diseases: a main challenge for medicine of the 21st century. Shanghai Arch Psychiatry.

[13] Zima BT, Rodean J, Hall M, Bardach NS, Coker TR, Berry JG (2016). Psychiatric disorders and trends in resource use in pediatric hospitals. Pediatrics.

[14] Perrin JM, Asarnow JR, Stancin T, Melek SP, Fritz GK (2019). Mental health conditions and health care payments for children with chronic medical conditions. Acad Pediatr.

[15] Lucas N, Bayer JK, Gold L, Mensah FK, Canterford L, Wake M (2013). The cost of healthcare for children with mental health difficulties. Aust N Z J Psychiatry.

[16] Knapp M, Scott S, Davies J (1999). The cost of antisocial behaviour in younger children. Clin Child Psychol Psychiatry.

[17] Romeo R, Knapp M, Scott S (2006). Economic cost of severe antisocial behaviour in children–and who pays it. Br J Psychiatry.

[18] (NICE) NIfhaCE (2019). Transforming mental health care for children and young people with long-term conditions: mental health and psychological wellbeing drop-in centre.

[19] Chiles JA, Lambert MJ, Hatch AL (1999). The impact of psychological interventions on medical cost offset: a meta-analytic review. Clin Psychol Sci Pract.

[20] Layard R, Clark DM (2015). Why more psychological therapy would cost nothing. Front Psychol.

[21] Kmietowicz Z. Increasing access to psychological therapies will cost NHS nothing, says report. BMJ 2012;344:e4250. 10.1136/bmj.e4250. 10.1136/bmj.e4250 22718919

[22] Jacobs K, Titman P, Edwards M (2012). Bridging psychological and physical health care. The Psychologist.

[23] Imran N, Zeshan M, Pervaiz Z (2020). Mental health considerations for children & adolescents in COVID-19 Pandemic. Pak J Med Sci.

[24] Jia R, Ayling K, Chalder T, Massey A, Broadbent E, Coupland C (2020). Mental health in the UK during the COVID-19 pandemic: cross-sectional analyses from a community cohort study. BMJ Open.

[25] NHS. Mental Health Service and COVID-19; Preparing for the rising tide. NHS RESET 2020 [Available from: https://www.nhsconfed.org/publications/mental-health-services-and-covid-19.

[26] Munblit D, Simpson F, Mabbitt J, Dunn-Galvin A, Semple C, Warner JO. Legacy of COVID-19 infection in children: long-COVID will have a lifelong health/economic impact. Arch Dis Child. 2022;107(3):e2. 10.1136/archdischild-2021-321882. Epub 2021 May 27. 10.1136/archdischild-2021-321882 34045207

[27] Ravens-Sieberer U, Kaman A, Erhart M, Devine J, Schlack R, Otto C. Impact of the COVID-19 pandemic on quality of life and mental health in children and adolescents in Germany. Eur Child Adolesc Psychiatry. 2021:1–11. 10.1007/s00787-021-01726-5. Epub ahead of print. 10.1007/s00787-021-01726-5 PMC7829493 33492480

[28] Crenna-Jennings W, Hutchinson J (2020). Access to child and adolescent mental health services in 2019.

[29] Catanzano M, Bennett SD, Kerry E, Liang H, Heyman I, Coughtrey AE (2021). Evaluation of a mental health drop-in centre offering brief transdiagnostic psychological assessment and treatment for children and adolescents with long-term physical conditions and their families: a single-arm, open, non-randomised trial. Evid Based Ment Health.

[30] Catanzano M, Bennett SD, Tibber MS, Coughtrey AE, Liang H, Heyman I (2021). A mental health drop-in centre offering brief transdiagnostic psychological assessment and treatment in a paediatric hospital setting: a one-year descriptive study. Int J Environ Res Public Health.

[31] Chorpita BF, Weisz JR (2009). Modular approach to therapy for children with anxiety, depression, trauma, or conduct problems (MATCH-ADTC).

[32] Sacristán JA, Abellán-Perpiñán J-M, Dilla T, Soto J, Oliva J (2020). Some reflections on the use of inappropriate comparators in CEA. Cost Eff Resour Alloc.

[33] (NICE) NIfhaCE (2012). The guidelines manual: 7.3 Economic evidence and guideline recommendations.

[34] (ONS) OfNS (2021). Consumer price inflation time series (MM23).

[35] (NICE) NIfhaCE (2020). CHTE methods review: task and finish group report - discounting.

[36] Goodman R (1997). The strengths and difficulties questionnaire: a research note. J Child Psychol Psychiatry.

[37] Varni JW, Seid M, Kurtin PS (2001). PedsQL™ 4.0: Reliability and validity of the Pediatric Quality of Life Inventory™ Version 4.0 Generic Core Scales in healthy and patient populations. Medical care..

[38] Krause KR, Bear HA, Edbrooke-Childs J, Wolpert M (2019). What outcomes count? Outcomes measured for adolescent depression between 2007 and 2017. J Am Acad Child Adolesc Psychiatry.

[39] Ye CY, Jeppson TC, Kleinmaus EM, Kliems HM, Schopp JM, Cox ED (2017). Outcomes that matter to teens with type 1 diabetes. Diabetes Educ.

[40] Flannery H, Jacob J (2020). Measuring psychological outcomes in paediatric settings: making outcomes meaningful using client-defined perspectives. Clin Child Psychol Psychiatry.

[41] Khan KA, Petrou S, Rivero-Arias O, Walters SJ, Boyle SE (2014). Mapping EQ-5D utility scores from the PedsQL generic core scales. Pharmacoeconomics.

[42] Weisz JR, Kuppens S, Eckshtain D, Ugueto AM, Hawley KM, Jensen-Doss A (2013). Performance of evidence-based youth psychotherapies compared with usual clinical care: a multilevel meta-analysis. JAMA Psychiat.

[43] (GOSH) GOSHfCNfT (2019). Annual Report and Accounts 2018–2019.

[44] (ONS) OfNS (2017). House prices: how much does one square metre cost in your area?.

[45] Marsden J (2015). House prices in London–an economic analysis of London’s housing market. Greater London Authority Economics.

[46] (UCL) UCL (2014). PWP Best Practice Guide; PWP Training Review.

[47] Employers N (2019). NHS Terms and Conditions (AfC) pay scales - including High Cost Area Supplement.

[48] NICE (2013). Guide to the methods of technology appraisal.

[49] McCabe C, Claxton K, Culyer AJ (2008). The NICE cost-effectiveness threshold: what it is and what that means. Pharmacoeconomics.

[50] Wiles NJ, Thomas L, Turner N, Garfield K, Kounali D, Campbell J (2016). Long-term effectiveness and cost-effectiveness of cognitive behavioural therapy as an adjunct to pharmacotherapy for treatment-resistant depression in primary care: follow-up of the CoBalT randomised controlled trial. Lancet Psychiatry.

[51] Herbert L, Hardy S (2019). Implementation of a mental health screening program in a pediatric tertiary care setting. Clin Pediatr (Phila).

[52] Bennett SD, Cuijpers P, Ebert DD, McKenzie Smith M, Coughtrey AE, Heyman I (2019). Practitioner review: unguided and guided self-help interventions for common mental health disorders in children and adolescents: a systematic review and meta-analysis. J Child Psychol Psychiatry.

[53] Baltussen R, Leidl R, Ament A (1999). Real world designs in economic evaluation. Bridging the gap between clinical research and policy-making. Pharmacoeconomics.

[54] Weisz JR (2015). Bridging the research-practice divide in youth psychotherapy: the deployment-focused model and transdiagnostic treatment. Verhaltenstherapie.

[55] Palmer S, Raftery J (1999). Economic notes: opportunity cost. BMJ.

