# Supplementary material for: Cost-effectiveness of a mental health drop-in centre for young people with long-term physical conditions
Source: BMC Health Serv Res. 2022 Apr 19;22:518. doi: 10.1186/s12913-022-07901-x (PMC9016208; doi:10.1186/s12913-022-07901-x)

**Supplementary Information:**

**Appendix A**: Patient Characteristics from the Outcome Study

|  | | **All participants (n= 186)** | |
| --- | --- | --- | --- |
|  | |  |  |
|  | |  | |
| Age of children and young people (<18 years) at the hospital in years, mean (SD) | 9 (4) | |  |
| Age of parents, mean (SD) | | 39 (8) | |
| IMD decile, median (IQR) | | 5 (3-9) | |
| Gender, % (n) | |  | |
| Female | | 62 (116/186) | |
| Male | | 38 (70/186) | |
| Primary recipient of the intervention, % (n/n total) | |  | |
| Patient | | 75 (139/186) | |
| Parent / carer | | 19 (36/186) | |
| Sibling | | 10 (18/186) | |
| Ethnicity, % (n/n total) | |  | |
| White | | 62 (116/186) | |
| Asian | | 11 (21/186) | |
| Black | | 10 (19/186) | |
| Any mixed background | | 7 (13/186) | |
| Any other ethnicity | | 4 (8/186) | |
| Not stated/prefer not to say | | 5 (9/186) | |
| Parent relationship to child, % (n/n total) | |  | |
| Mother | | 90 (167/186) | |
| Father | | 10 (19/186) | |
| Parent marital status, % (n/n total) | |  | |
| Married | | 60 (62/104) | |
| Single | | 16 (17/104) | |
| Divorced/separated | | 12 (12/104) | |
| Living with partner | | 9 (9/104) | |
| Widowed | | 1 (1/104) | |
| Not stated/prefer not to say | | 3 (3/104) | |
| Parent employment status, % (n/n total) | |  | |
| Employed (full time) | | 30 (31/104) | |
| Employed (part time) | | 24 (25/104) | |
| Other | | 18 (19/104) | |
| Out of work | | 11 (11/104) | |
| Self-employed | | 8 (8/104) | |
| Unable to work | | 6 (6/104) | |
| Retired | | 1 (1/104) | |
| Not stated/prefer not to say | | 3 (3/104) | |
| Parent disability, % (n/n total) | |  | |
| Yes | | 11 (11/104) | |
| No | | 89 (93/104) | |
| Presenting problems, % (n/n total) | |  | |
| Anxiety | | 45 (84/186) | |
| Challenging behaviour | | 38 (70/186) | |
| Low mood | | 28 (52/186) | |
| Other | | 14 (26/186) | |
| Known pre-existing neurodevelopmental diagnosis, % (n/n total) | |  | |
| Autism Spectrum Disorder | | 15 (27/186) | |
| Intellectual Disability | | 21 (39/186) | |
| None | | 62 (115/186) | |
| Not stated/prefer not to say | | 4 (7/186) | |
| Patient type, % (n/n total) | |  | |
| Outpatient | | 96 (100/104) | |
| Inpatient | | 4 (4/104) | |
| Need for translator, % (n/n total) | |  | |
| Yes | | 4 (8/186) | |
| No | | 96 (178/186) | |
| County of origin, % (n/n total) | |  | |
| <50 miles of London | | 81 (151/186) | |
| >50 miles of London | | 15 (27/186) | |
| Not stated/prefer not to say | | 4 (8/186) | |
| History of mental health input, % (n/n total) | |  | |
| Yes | | 46 (86/186) | |
| No | | 53 (98/186) | |
| Not stated/prefer not to say | | 1 (2/186) | |
| History of risk present, % (n/n total) | |  | |
| Yes | | 18 (34/186) | |
| No | | 81 (151/186) | |
| Not stated/prefer not to say | | 1 (1/186) | |
| Primary intervention allocated to, % (n/n total) | |  | |
| MATCH | | 32 (59/186) | |
| Referral | | 45 (84/186) | |
| Neurodevelopmental assessment | | 3 (6/186) | |
| Signposting to resources only | | 19 (35/186) | |
| Other | | 1 (2/186) | |
| Self-reported change in physical health (6 months post), % (n/n total) | |  | |
| Improved | | 10 (10/104) | |
| No change | | 40 (42/104) | |
| Deteriorated | | 6 (6/104) | |
| Not stated | | 44 (46/104) | |

*NB: where total sample size is 104, this means data was only collected in the second year of the trial (post-pilot phase); IMD decile=Index of multiple deprivation decile.*

**Appendix B**: Base-case Model ICER Scatter Plot


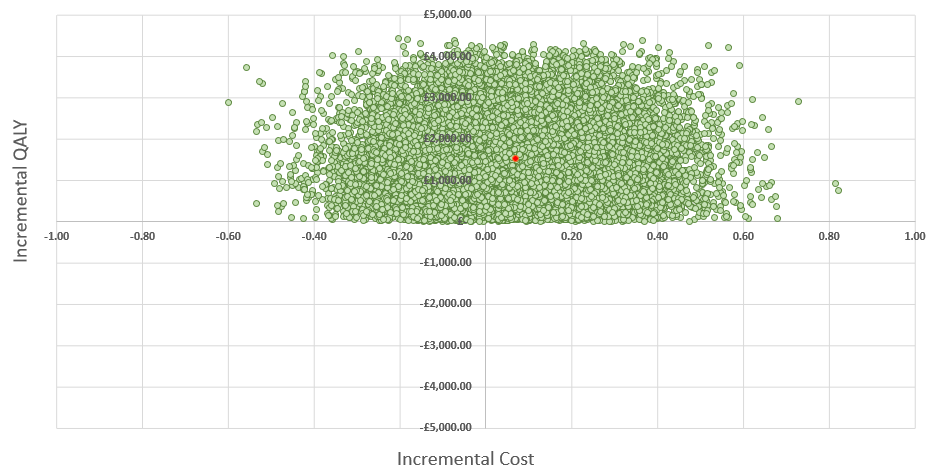


**Appendix C**: Practical Model ICER Scatter Plot


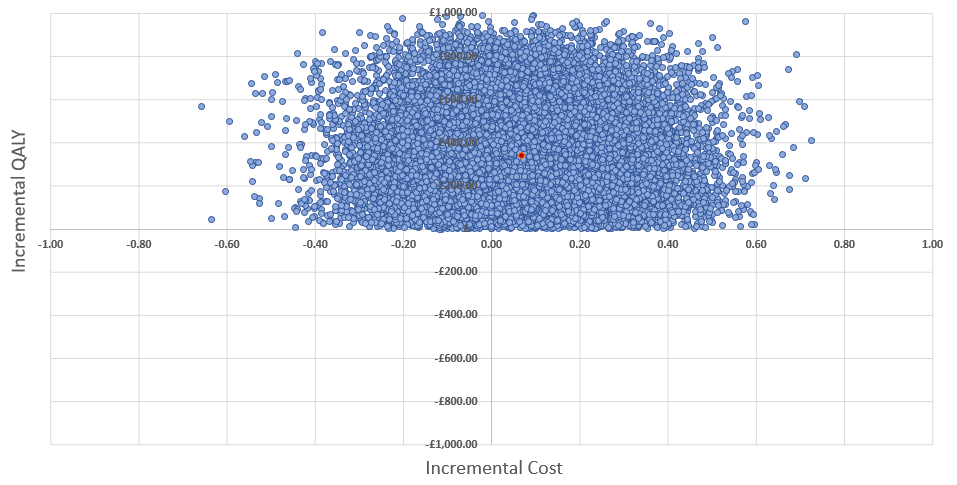

Supplement: Supplementary file 1 — Additional file 1: Appendix A. Patient Characteristics from the OutcomeStudy. Appendix B. Base-case Model ICER Scatter Plot. Appendix C. Practical Model ICER Scatter Plot. [file 12913_2022_7901_MOESM1_ESM.docx]
